# Subunit-specific mechanisms of isoflurane-induced acute tonic inhibition in dentate gyrus granule neuron

**DOI:** 10.3389/ebm.2024.10171

**Published:** 2024-10-28

**Authors:** Zhiqiang Yu, Xiaodan Chen, Zheng Liu, Ran Ding, Jin Xu

**Affiliations:** ^1^ Department of Anesthesiology, Tianjin Central Hospital for Gynecology and Obstetrics, Tianjin, China; ^2^ Tianjin Research Institute of Anesthesiology and Department of Anesthesiology, Tianjin Medical University General Hospital, Tianjin, China; ^3^ Department of Anesthesiology, Tianjin Hospital, Tianjin, China

**Keywords:** dentate gyrus, isoflurane, tonic current, GABAA receptor, knockout

## Abstract

Prolonged exposure to volatile anesthetics may raise the risk of developing cognitive impairment by acting on gamma-a Aminobutyric acid A receptors (GABAAR). The dentate gyrus plays an important role in the hippocampus and has a high potential for neural plasticity. However, it is unknown whether prolonged anesthesia induces a change in acute phasic or tonic inhibition in dentate gyrus granule cells (DGGCs) by acting on GABAAR. In order to verify the effects of volatile anesthetics on the current in DGGCs, a whole-cell patch was applied to record acute brain slices, and this study indicated that 4 h but not 2 h of isoflurane (ISO) exposure induced significantly larger tonic currents in DGGCs other than hippocampal CA1 pyramidal and thalamic relay neurons. Furthermore, this study demonstrated that the increased tonic current in DGGCs was dependent on the δ subunit-containing GABAARs by using transgenic δ subunit knockout mice. In conclusion, the δ subunit specific GABAAR is the key element that increased acute tonic inhibition in DGGCs of mice after prolonged ISO exposure, which may be one of the mechanisms of ISO neurotoxicity to the developing brain.

## Impact statement

To verify the effect of long-term general anesthesia on neurotoxicity by studying the subunit specific mechanism of acute ankylosing inhibition of dentate gyrus granule neurons induced by isoflurane.

## Introduction

Millions of surgical procedures are performed annually under general anesthesia. There is a growing concern that prolonged general anesthetic exposure might result in brain disorders in young children and geriatric patients and increase the risk of cognitive impairments [1, 2]. Although conclusive evidence of general anesthetic neurotoxicity in humans has not yet been confirmed, many animal researches have indicated that exposure to general anesthetics during early postnatal life impairs neurocognitive function. The cellular and molecular mechanisms underlying this potential hazard of general anesthetics remain poorly understood.

The mechanism by which general anesthetics impair cognition may involve a decrease in hippocampal neurogenesis [3, 4]. Rodent models have shown that alterations in hippocampal neurogenesis induced by volatile anesthetics contribute to brain impairment, such as numerous deficits in learning and memory [4–6]. Postnatally, learning, memory, and cognitive ability are closely correlated with neurogenesis and neurodevelopment, especially in newly generated mature dentate gyrus granule cells (DGCs) [7]. Considerable evidence from rodents and nonhuman primates has demonstrated that the neurogenesis of granule neurons in the dentate gyrus (DG) can be influenced by volatile anesthetics such as isoflurane (ISO) and sevoflurane [8–12]. However, the mechanism by which volatile anesthetics affect the neurogenesis of granular neurons in the DG remains unclear.

GABA type A receptors (GABAARs) generate rapid inhibitory neuronal transmission and are critical for regulating memory, mood, sleep, and nervous system excitability. GABAARs mediate two different types of inhibition: phasic inhibition mediated by postsynaptic receptors and tonic inhibition mediated by extrasynaptic receptors located outside the synapse [13, 14], which induces in variations in GABAergic conductance across different brain areas and neuron types [15]. In the past 20 years, the relationship between dysfunctional GABAergic system and the neurotoxicity of volatile anesthetics is one of the most explored topics. Tonic inhibition and phasic inhibition are crucial for maintaining a balance between the inhibitory and excitatory systems. Volatile general anesthetics are positive allosteric modulators (PAMs) of GABAARs and are widely used for analgesia and sedation. ISO is one of the most widely used volatile anesthetics. Based on current evidence, prolonged exposure to ISO [16, 17] has been confirmed as a possible risk factor in interrupting the function of dentate gyrus granule cells (DGGCs) [3, 18–20]. However, whether prolonged ISO exposure induces a change in acute phasic inhibition and tonic inhibition in DGGCs by acting on GABAAR remains unclear.

Further more, γ2-subunit predominantly contribute to phasic GABAAR-mediated currents in DGCs, α5 and δ subunits predominantly contribute to tonic GABAAR-mediated currents in DGCs. In order to verify the effects of volatile anesthetics on phasic and tonic currents, and observe the effects of volatile anesthetics on the currents dependent on γ2, α5 and δ subunit-containing GABAARs, we used a whole-cell patch to observe the effects of ISO exposure on acute phasic inhibition and tonic inhibition in DGGCs. Specific antagonists of the subunits and transgenic subunit knockout mice are used to observe the effects of ISO exposure on the γ2, α5 and δ subunit-containing GABAARs currents in DGGCs.

## Materials and methods

### Mice

The use of animals in all experiments were approved by Animal Ethics Committee of Tianjin Central Hospital of Gynecology and Obstetrics according to the National Institutes of Health guidelines. Male or female mice are both suitable to use, and male mice were used in this study. C57BL/6 (Beijing Weitonglihua Experimental Animal Technology Co., Ltd; Beijing; China) and Gabrd KO mice, 3 weeks, 10–15 g, were used in this study. The Gabrd KO mice were shared by professor Hui Shen of Tianjin Medical University. The Gabrd KO mice were derived from C57BL/6 mice, and the mice have been backcrossed to C57BL/6J for ten generations. The mice were weaned and genotyped at postnatal day 21 (P21). Quantitative real-time PCR was used to analyze the transcriptional profile of the GABAAR subunits, and the information of sequences of the primers were provided in the Supplementary Material. To minimize bias, the birth time are similar, the mice age, gender and feeding environment are same between Control group (C57BL/6) and Gabrd KO mice group. All mice were bred in the temperature-fixed, humidity-controlled animal colony with a 12 h light/dark circle (7:00 a.m. to 7:00 p.m.). All mice used in the experiments were housed in several cages, and provided with sufficient food and water throughout the study.

### Exposure of mice to ISO

The mice were randomly divided to the following three groups: Control group (no ISO exposure, Control), ISO 2 h group (exposed to 1.5% ISO for 2 h, ISO 2 h), and ISO 4 h group (exposed to 1.5% ISO for 4 h, ISO 4 h). ISO was delivered using a pour**-**fill vaporizer group (RWD R500), whereas the animals in the Control group were exposed to 21% O_2_ for 30 min. During exposure to ISO, the chamber temperature was kept at approximately 39°C with a custom-made heating pad. The respiration was monitored visually, and the rectal temperature of mice was monitored and maintained between 36.5 and 37.5°C throughout the experiments.

### Electrophysiological recording

Whole-cell voltage-clamp recordings were recorded in the DGGCs in the Control, ISO 2 h, and ISO 4 h groups. Mouse hippocampal and thalamic slices were prepared as previous study described [21]. Briefly, animals were quickly decapitated under ISO anesthesia, and the brains were dissected in ice and oxygenated artificial cerebrospinal fluid (ACSF) containing 125 mM NaCl, 4.5 mM KCl, 1.25 mM NaH_2_PO_4,_ 26 mM NaHCO_3_, 1 mM MgCl_2,_ 2 mM CaCl_2_, and 20 mM glucose (pH 7.4 when bubbled with 95% oxygen and 5% CO_2_). Coronal slices (350–380 µm) containing the bilateral hippocampus CA1/DG and thalamus were prepared incubated at 34°C in an oxygenated sucrose-based ACSF for at least 45 min and subsequently, all slices were kept at room temperature to recover for another 30 min before performing whole-cell voltage-clamp. A single slice was placed in a recording chamber, where it was continuously perfused (2 mL min/L) with ACSF saturated with 95% O_2_/5% CO_2_ at room temperature, which was placed on the fixed stage of an upright Olympus BX50WI microscope (Olympus, Tokyo, Janpan). The currents in acute cortical slices of pyramidal neurons in the hippocampal CA1/DG and thalamic relay neurons were recorded by Whole-cell patch-clamp.

The recording glass pipettes with resistance 4–6 MΩ were full of an intracellular solution, which containing: 10 mM HEPES, 130 mM CsCl, 5 mM QX314, 8 mM NaCl, 0.3 mM Na-GTP, 4 mM Mg-ATP, and 0.2 mM EGTA; pH: 7.3. To record and observe the tonic GABAAR currents, 50 μM DNQX, 5 μM CGP 52432 and 50 μM APV were regularly added into the bath electrolyte to block the B type GABA receptors and ionotropic glutamate. To reveal the total tonic GABA current, local perfusion of 10 μM bicuculline (BIC, sigma) was used to block all GABAA-Rs and change the holding current. To reveal the tonic current mediated by α5-containing GABAARs, local application of 100 nM L-655,708 (Tocris) was used, which is a selective inverse agonist for α5 GABAARs. The membrane potential was kept at −70 mV. Spontaneous miniature inhibitory postsynaptic currents (mIPSCs) were pharmacologically separated by bath application of NBQX (50 µM), D-AP5 (50 µM), and TTX (1 µM), and all slices were measured at −60 mV of voltage clamp mode. Access resistance was regulated before and after each recording. Series resistance was typically 10–20 MΩ. Neurons were abandoned when the parameter was higher than 30 MΩ or varied more than 20%. An Axon 700B amplifier (Molecular Devices, LLC., San Jose, CA, United States) was applied to record electrical signals. All data were filtered at 10 kHz and digitized at 20 kHz using a Digidata-1550B system with clampfit 10.6 software (Molecular Devices, LLC., San Jose, CA, United States).

### Data analysis and statistics

Tonic GABAAR currents were defined as the amplitude change following BIC perfusion and measured as previous studies described [22, 23]. The frequency and amplitude of sIPSCs were analyzed offline using clampfit 10.6 (Molecular Devices, LLC., San Jose, CA, United States) and GraphPad Prism software v6. Differences of the tonic currents and the sIPSC parameters were analyzed by one-way analysis of variance (ANOVA) followed by Sidak *post hoc* t-test. Unless otherwise stated, data were represented as mean ± standard deviation. An unpaired t-test was applied to the independent samples. A P-value of <0.05 was considered that the difference was statistically significant.

## Results

### Prolonged ISO exposure induced the tonic GABAAR current in DGGCs

**FIGURE 1:**
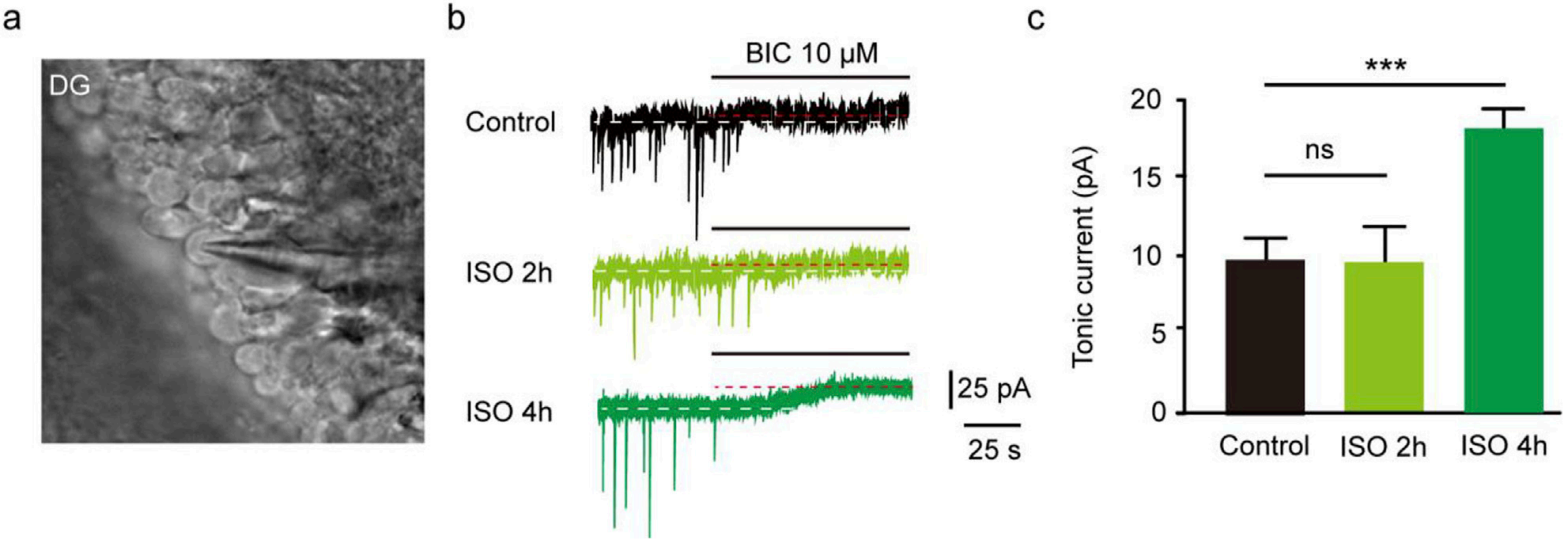
Exposed ISO for 4 h induced tonic current in DGGCs. **(A)** Image of brain slice. **(B)** Samples of the tonic current recording in Control, ISO 2 h, and ISO 4 h group. **(C)** Mean change in tonic current in Control, ISO 2 h and ISO 4 h group (Control, 8.75 ± 0.75 pA, n = 6 cells, one cell per mouse; ISO 2 h, 8.42 ± 1.04 pA, n = 5 cells, one cell per mouse; ISO 4 h, 17.90 ± 1.01 pA, n = 5 cells, one cell per mouse; one-way ANOVA, *F* [2, 13] = 64.67, ***p < 0.001, ns, not significant).

**FIGURE 2:**
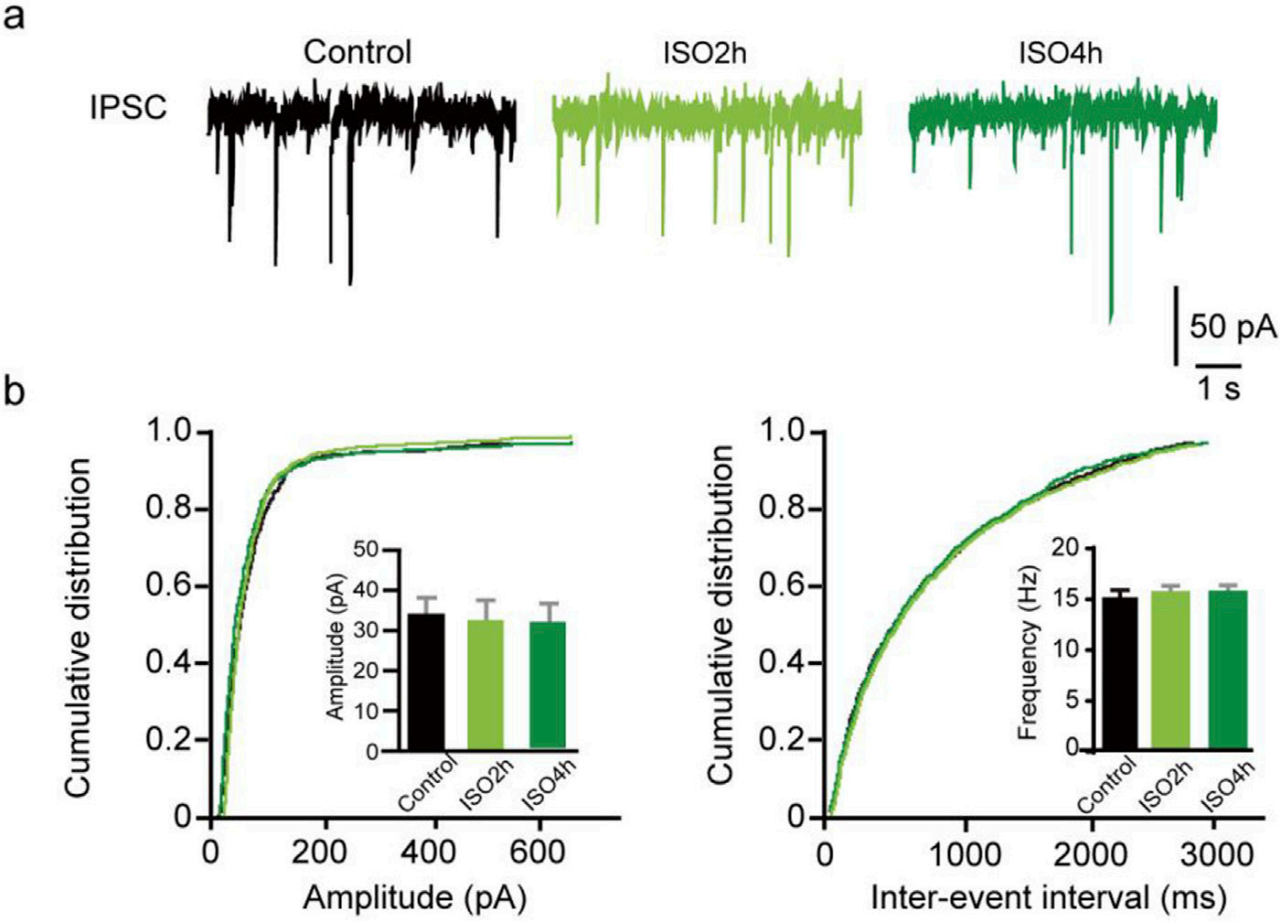
ISO do not change the phasic mIPSC frequency and amplitude in DGGCs. **(A)** Representative traces of mIPSCs recorded from dentate granule cells of adult mice. **(B)** Quantifications of mIPSC amplitude and cumulative distributions of the inter-event intervals. mIPSC amplitudes are comparable between Control and ISO group (Control, 31.06 ± 4.64 pA, n = 11 cells, one cell per mouse; ISO 2h, 32.85 ± 4.31 pA, n = 10 cells, one cell per mouse; ISO 4h, 35.84 ± 5.65 pA; n = 10 cells, one cell per mouse; one-way ANOVA, *F* [2, 28] = 2.532, *P* = 0.10). Quantifications of mIPSC frequency and cumulative distributions of the mIPSC inter-event interval. mIPSC inter-event interval are comparable between control and ISO group (Control, 16.45 ± 4.05 Hz, n = 11 cells, one cell per mouse; ISO 2h, 20.44 ± 3.82 Hz, n = 10 cells, one cell per mouse; ISO 4h, 18.30 ± 3.74 Hz, n = 10 cells, one cell per mouse; one-way ANOVA, *F* [2, 28] = 2.774, *p* = 0.08).

The results revealed that the tonic currents of the ISO 2 h group were comparable to those of the Control group (*P =* 0.84, Figures 1A–C). However, in the ISO 4 h group, tonic GABAAR currents were significantly larger than those of the Control group and ISO 2 h group (*F* [2, 13] = 64.67, *P* < 0.001; Figures 1B, C). Next, to investigate whether other brain regions exhibited enhanced tonic GABAAR currents following ISO exposure, hippocampal CA1 pyramidal neurons and thalamic relay neurons were recorded by whole-cell voltage-clamp under the same conditions as the DGGCs. The tonic GABAAR currents in the two brain areas were not altered by ISO exposure (*F* [2, 28] = 2.532, *P* = 0.10; *F* [2, 28] = 2.774, *P* = 0.08; Figures 2A, B), implying that ISO exposure preferentially increased tonic GABAA receptor currents in the DG but not in other examined brain regions. These results suggest that acute inhibitory tonic GABAAR currents were time-and location-dependent in DGGCs.

### Prolonged ISO exposure did not change the phasic inhibitory postsynaptic transmission

**FIGURE 3:**
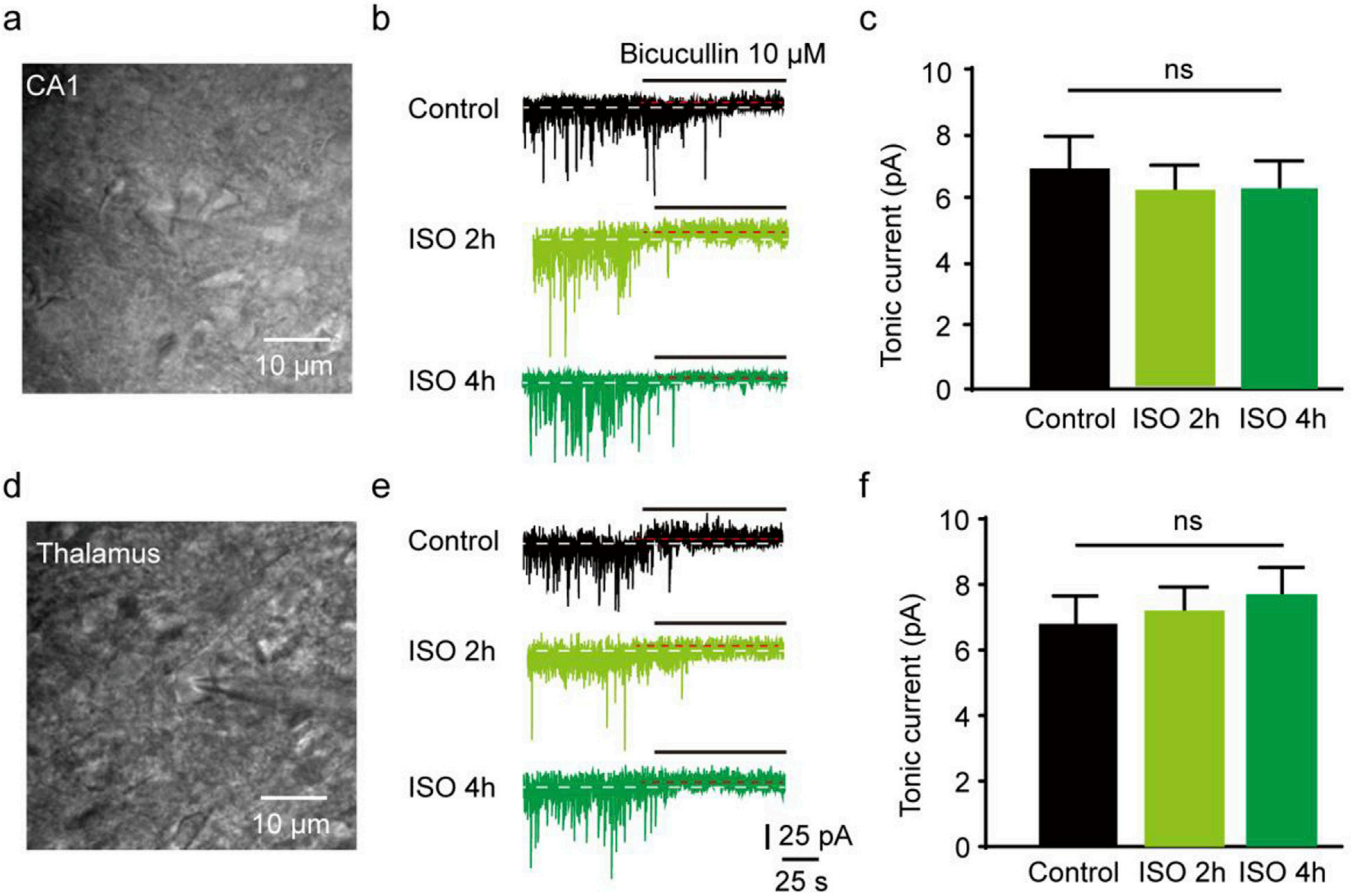
Hippocampus CA1 and thalamus relay cells showed no enhanced tonic current by 2 and 4 h ISO exposure revealed by GABAAR antagonist BIC, compared with Control group respectively. **(A)** Example of image, hippocampus CA1 pyramidal neuron. **(B)** Samples of the tonic current recording from CA1 pyramidal neuron. **(C)** The average amplitude of tonic current (pA) in Control, ISO 2h, and ISO 4 h group (Control, 6.89 ± 0.72 pA, n = 7 cells, one cell per mouse; ISO 2h, 6.11 ± 0.70 pA, n = 8 cells, one cell per mouse; ISO 4h, 6.23 ± 0.75 pA, n = 8 cells, one cell per mouse, one-way ANOVA, *F* [2, 20] = 2.44, *p* = 0.11). **(D)** Example of image, thalamus relay neuron, scale bar 10 μM. **(E)** Samples of the tonic current recording from thalamus relay neurons. **(F)** The average amplitude of tonic current (pA) in Control, ISO 2h, and ISO 4 h group (Control, 6.87 ± 0.72 pA, n = 7 cells, one cell per mouse; ISO 2h, 7.19 ± 0.65 pA, n = 8 cells, one cell per mouse; ISO 4h, 7.56 ± 0.64 pA, n = 8 cells, one cell per mouse; one-way ANOVA, *F* [2, 20] = 2.01, *p* = 0.16).

Furthermore, whole-cell voltage-clamp recordings were used to determine whether ISO exposure changed the basal phasic postsynaptic inhibitory transmission in DGGCs. After blocking the excitatory postsynaptic currents and action potentials, mIPSCs were isolated and recorded from DGGCs of juvenile animals. A variation in mIPSC amplitude suggests a variation in the number of ionic GABAA receptors at postsynaptic sites, whereas a variation in mIPSC frequency implies a change in the synapse number or presynaptic vesicle release odds. There was no difference in the mIPSC amplitude in the Control, ISO 2h, and ISO 4 h groups, suggesting 4 h ISO exposure does not regulate the postsynaptic GABAA receptor number (*F* [2, 20] = 2.44, *P* = 0.11; Figures 3A–C). There was no obvious difference in mIPSC frequency among the control, ISO 2h, and ISO 4 h groups (*F* [2, 20] = 2.01, *P* = 0.16; Figures 3D–F). These results demonstrated that 2 h and 4 h of ISO exposure did not change the phasic postsynaptic inhibitory transmission in dentate granule cells.

### Prolonged ISO exposure induced acute tonic current dependent on δ other than α5 subunit-containing GABAA receptor

**FIGURE 4:**
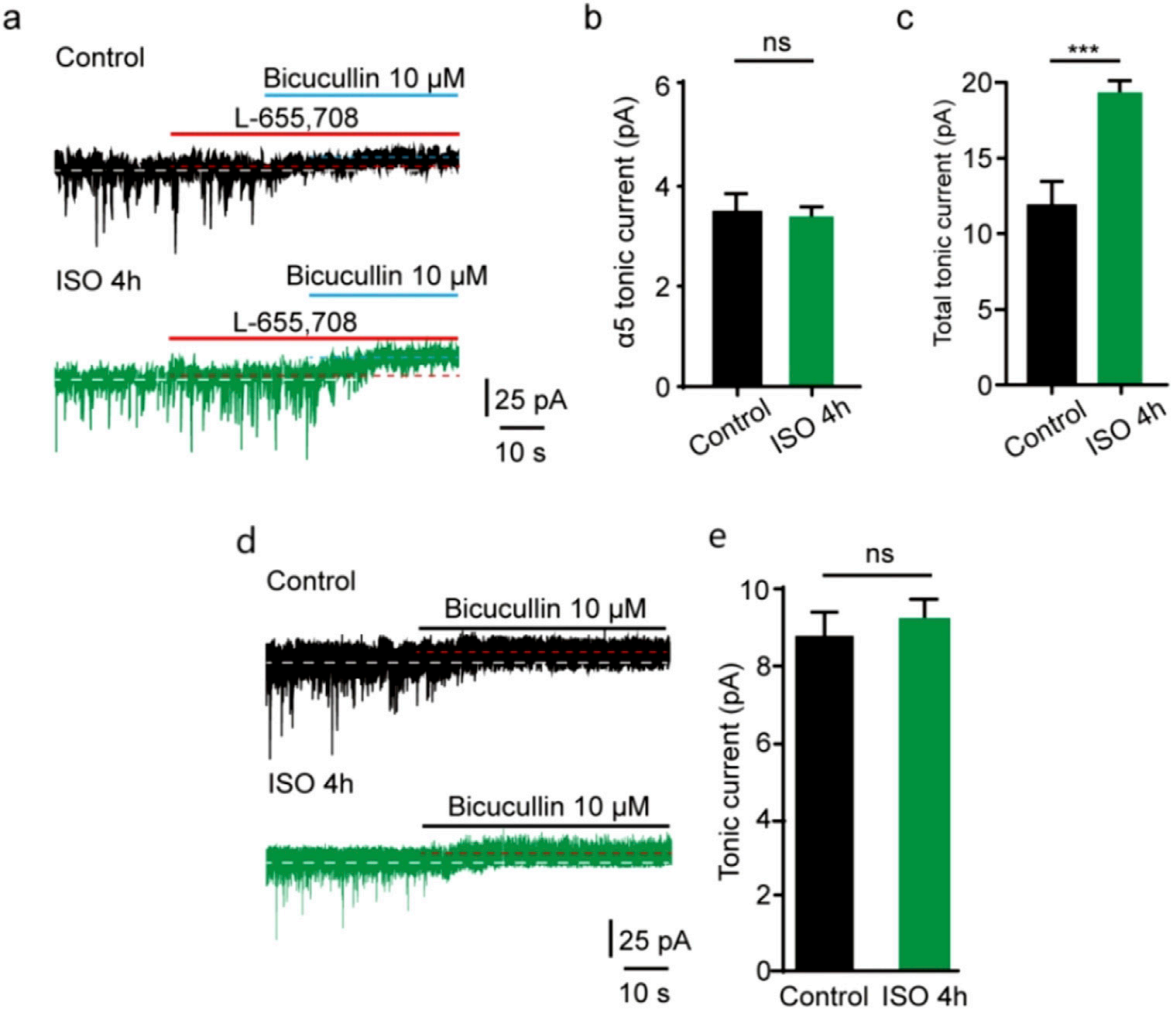
ISO-induced tonic current did not change by α5 subunit antagonist. **(A)** Representative tonic current recordings (Up, Control; Middle, ISO 4 h; Down, ISO 4 h + α5 subunit antagonist). **(B)** Statistics of the average amplitude tonic current by α5 subunit antagonist L-665,708 (Control, 3.51 ± 0.53 pA, n = 7 cells, one cell per mouse; ISO 4 h, 3.11 ± 0.36 pA, n = 7 cells, one cell per mouse; Unpaired t-test, t = 1.64, df = 12, *p =* 0.13) **(C)** The average amplitude of total tonic current (pA) in control and ISO 4 h group (Control, 12.23 ± 1.46 pA, n = 7 cells, one cell per mouse; ISO 4h, 19.21 ± 1.60 pA, n = 7 cells, one cell per mouse; Unpaired t-test, t = 8.52, df = 12, ***p < 0.001. ns, not significant). **(D)** Samples of tonic current. Up, control; Down, 4 h ISO pretreatment. **(E)** Mean change in tonic current (pA) in control and ISO 4 h group (Control, 8.80 ± 0.83 pA, n = 7 cells, one cell per mouse; ISO 4h, 9.70 ± 1.30 pA, n = 7 cells, one cell per mouse; Unpaired t-test, t = 1.54, df = 12, Control vs. ISO 4h, *p* = 0.15).

In the CNS, some subunits exhibit restricted expression profiles. α5 subunit is abundantly expressed in the hippocampus and DG [14, 24], and the excessive function of extrasynaptic α5-containing GABAAR after general anesthesia has been found [25]. δ subunits are also located at extrasynaptic sites where GABAAR are activated by ambient GABA [13, 14, 26]. To examine whether extrasynaptic α5-containing GABAAR was associated with the acute tonic current induced by prolonged ISO exposure, a selective inverse agonist for α5 GABAARs (L-655,708) was used. Our study showed that the tonic current blocked by L-655,708 in the control group was comparable to that in the ISO 4 h group in DGGCs (t = 1.64, df = 12, *P=* 0.13; Figures 4A, B). Meanwhile, the total tonic current blocked by BIC in the ISO 4 h group was nearly double that in the control group (t = 8.52, df = 12, *P* < 0.001; Figures 4A, C), which is consistent with the previous values (Figure 1C). However, up to now, there was no suitable δ subunit GABAAR antagonist; the tonic currents were recorded in δ subunit knockout mice in DGGCs. Identified with our prediction, using the δ subunit knockout mice, we found tonic inhibition currents comparable to the control group (t = 1.54, df = 12, *P* = 0.15; Figures 4D, E).

## Discussion

We found that prolonged ISO exposure induced larger tonic GABAAR currents in DGGCs, but not in the hippocampal CA1 and thalamus. Additionally, prolonged ISO exposure did not change the phasic mIPSC frequency and amplitude in DGGCs. Meanwhile, we indicated that the increased tonic current after prolonged ISO exposure in the DGGCs was mediated by the δ subunit-containing GABAARs.

### Prolonged ISO exposure increased acute tonic current in DGGCs

As key mediators of inhibitory signals in the brain, GABAARs comprise dozens of highly heterogeneous subtypes regarding their pharmacological properties, subunits and subcellular location [27]. Among these receptors, some GABAARs are known to be located postsynaptically and extrasynaptically and account for phasic and tonic inhibition in many different brain regions. This study discovered that the tonic current in DGGCs was significantly increased when ISO exposure was up to 4 h but not 2 h. However, ISO exposure did not change phasic inhibitory postsynaptic transmission in DGGCs. Our results showed that 4 h of ISO exposure did not increase the tonic current in the hippocampal CA1 or thalamus. δ-containing GABAARs tonic currents contribute critically to inhibition in DGGCs but not in hippocampal CA1 and thalamic relay neurons.

### Prolonged ISO exposure increased acute tonic current dependent on δ other than α5 subunit-containing GABAA receptor

Tonic inhibition regulates neuronal excitability, δ-GABARs are localized to the extrasynaptic and perisynaptic membrane of DGCs, and the tonic GABAAR current in DGGCs is mainly mediated by δ subunit-containing receptors [28]. Low ambient GABA levels in the extracellular space could generate tonic inhibition in DG that expressed δ-containing GABAARs. The α5-containing GABAARs that make up 25% of the receptor subtype are located in the learning and memory-related region of the brain. In addition to exhibiting synaptic phasic inhibition, the extrasynaptic α5-containing receptors mediate tonic inhibition [26, 29]. The tonic inhibition that mediated by α5-containing GABAAR in the DG plays an significant role in cognitive function [30–32].

In this study, prolonged ISO exposure induced larger tonic GABAAR currents in DGGCs, and the amplitude values of tonic inhibition with the α5 subunit antagonist L-655,708 [33] were comparable to the 4 h ISO exposure group, which indicated that the increased tonic current was not dependent on the α5 subunit-containing GABAARs. Furthermore, whether the increased tonic current induced by prolonged ISO exposure depended on the δ subunit-containing GABAARs in DGGCs was considered in this study. Although many drugs selectively enhance-unk-containing GABAARs, no specific blocker can be used. We exposed δ subunit knockout mice to ISO for 4 h and found that tonic current values were comparable to the control levels. Hence, our findings have thus far shown that the δ-containing GABAARs have key roles in contributing to DGGCs tonic inhibition by ISO exposure.

### Effects of the increased acute tonic current in DGGCs on neuronal development and cognitive function

The DG is the main entrance to the hippocampus and plays a important role in emotion, learning, and memory. The DG, as an important part of the hippocampus, has a high potential for neural plasticity, and the maturation and neurogenesis of DGCs in the postnatal life in the subgranular zone is conducive to the capability of the DG to transform [34]. Therefore, factors that potentially affect the development and maturation of neurons in the DG may have long-lasting effects on brain function. Some studies have indicated the effects of general anesthetics exposure on cognitive function; one common feature is that anesthesia exposure disrupts the generation and development of DGCs in the hippocampus [7, 35, 36]. A previous study demonstrated that hippocampal DGCs are very vulnerable to ISO-induced neurotoxicity in juvenile mice [37]. Tonic inhibition of DGGCs negatively regulates neuronal development and neurocognitive function. We found that the tonic current in DGGCs was increased by prolonged ISO exposure. Hence, we inferred that prolonged ISO exposure may impair neuronal development and cognitive function by increasing the acute tonic current in DGGCs.

δ-containing GABAARs paly an important role in inhibitory network in mediating acute brain impairment, and δ receptors have been defined are associated with consciousness, mood disorders, epilepsy, and schizophrenia. Tonic inhibitory current is generated by activating extrasynaptic GABAA receptors. δ subunit-containing receptors of GABAAR mediate tonic current in DGGCs, which inhibits learning and memory and modulates the function of DGCs. By pharmacologically isolating δ-containing receptors, this study demonstrated that tonically activated δ-containing GABAARs are strongly modulated by prolonged ISO exposure because of their contribution to IPSCs in DGCs. Hence, prolonged ISO exposure may impair neuronal development and cognitive function by increasing the acute tonic current dependent on δ subunit specific GABAAR in DGGCs.

This study had a few limitations. First, there are several subunit-containing receptors of GABAAR-mediated tonic current in DGGCs, but we only selected δ and α5, two common subunit-containing receptors, to observe the effects of prolonged ISO exposure on tonic current in DGGCs. Second, we only observed an acute tonic current in the DGGCs but did not observe whether the increased tonic current was persistent. Third, we did not evaluate the cognitive function of the mice using long-term potentiation or water maze. Fourth, the knockout and control mice were derived from distinct litters in the present study, which may induce potential bias due to differences in genetic background, fetal neurodevelopment, and/or maternal behaviors between the two groups. Therefore, further studies are warranted.

## Conclusion

Our data suggest that δ subunit specific GABAAR is the key element of the acute tonic inhibition in juvenile mice in DGGCs by prolonged ISO exposure.

## Data Availability

The datasets presented in this study can be found in online repositories. The names of the repository/repositories and accession number(s) can be found in the article/Supplementary Material.
