# Supplementary material for: Subunit-specific mechanisms of isoflurane-induced acute tonic inhibition in dentate gyrus granule neuron
Source: Exp Biol Med (Maywood). 2024 Oct 28;249:10171. doi: 10.3389/ebm.2024.10171 (PMC11550974; doi:10.3389/ebm.2024.10171)
Supplement: Supplementary file 1 [file Table1.docx]

Table1. Sequences of the primers used in qRT-PCR

| Gene | Direction | Sequence |
| --- | --- | --- |
| *Gabra1* | Forward | 5’-AAAAGTCGGGGTCTCTCTGAC-3’ |
|  | Reverse | 5’-CAGTCGGTCCAAAATTCTTGTGA-3’ |
| *Gabra2* | Forward | 5’-AGAAAAACCCTCTTCTTCGGATG-3’ |
|  | Reverse | 5’-GTGGCATTGTTCATTTGAATGGT-3’ |
| *Gabra3* | Forward | 5’-ATGGGCACTTTTATGTGACCA-3’ |
|  | Reverse | 5’-CCCCAGGTTCTTGTCGTCTTG-3’ |
| *Gabra4* | Forward | 5’-ACAATGAGACTCACCATAAGTGC-3’ |
|  | Reverse | 5’-GGCCTTTGGTCCAGGTGTAG-3’ |
| *Gabra5* | Forward | 5’-TGACCCAAACCCTCCTTGTCT-3’ |
|  | Reverse | 5’-GTGATGTTGTCATTGGTCTCGT-3’ |
| *Gabrb1* | Forward | 5’-TCCCGTGATGGTTGCTATGG-3’ |
|  | Reverse | 5’-CCGCAAGCGAATGTCATATCC-3’ |
| *Gabrb2* | Forward | 5’-ATGTCGCTGGTTAAAGAGACG-3’ |
|  | Reverse | 5’-CTGCCACTCGGTTGTCCAAA-3’ |
| *Gabrb3* | Forward | 5’-CACGCTTGACAATCGAGTGG-3’ |
|  | Reverse | 5’-GCGGATCATGCGGTTTTTCAC-3’ |
| *Gabrg2* | Forward | 5’-AGAAAAACCCTCTTCTTCGGATG-3’ |
|  | Reverse | 5’-GTGGCATTGTTCATTTGAATGGT-3’ |
| *Gabrd* | Forward | 5’-ATTGGGGACTACGTGGGCT-3’ |
|  | Reverse | 5’-CCACATTCACAGGAGCACC-3’ |
| *Gabrr1* | Forward | 5’-CGAGGAGCACACGACGATG-3’ |
|  | Reverse | 5’-GTGAAGTCCATGTCAACCTCTG-3’ |
| *Gapdh* | Forward | 5’-AGGTCGGTGTGAACGGATTTG-3’ |
|  | Reverse | 5’-TGTAGACCATGTAGTTGAGGTCA-3’ |

Table 2. Sequences of shRNAs targeting *Gabrd* are provided as below

| Name of shRNA | Sequence |
| --- | --- |
| shRNA1(*Gabrd*) | CCAGGGCAATGAATGACAT |
| shRNA2(*Gabrd*) | CCACGGAGCTGATGAACTT |
| shRNA3(*Gabrd*) | GGAAGAAACGGAAAGCCAA |
